# Perceived weight gain and eating disorder symptoms among LGBTQ+ adults during the COVID-19 pandemic: a convergent mixed-method study

**DOI:** 10.1186/s40337-021-00470-0

**Published:** 2021-09-16

**Authors:** Jennifer Tabler, Rachel M. Schmitz, Ruby Charak, Eliza Dickinson

**Affiliations:** 1 Department of Criminal Justice and Sociology, University of Wyoming, 1000 E University Ave, Laramie, WY 82070 USA; 2 Department of Sociology, Oklahoma State University, Stillwater, OK USA; 3 Department of Psychological Science, The University of Texas Rio Grande Valley, Edinburg, TX USA; 4 Department of Criminal Justice and Sociology, Department of Zoology and Physiology, University of Wyoming, Laramie, WY USA

**Keywords:** LGBTQ, COVID-19, Weight gain, EDE-QS

## Abstract

**Background:**

In this study, we further explore the role of COVID-19 pandemic-related stress, social support, and resilience on self-reported eating disorder symptoms (using the EDE-QS) and perceived weight gain among lesbian, gay, bisexual, transgender, and/or queer (LGBTQ+ adults) in the US context during the COVID-19 pandemic.

**Methods:**

Employing a convergent mixed method design, we surveyed 411 individuals, and conducted qualitative semi-structured follow-up interviews with 43 LGBTQ+ -identifying survey respondents. Using OLS regression and multinomial logistic regression, we modeled eating disorder symptoms and perceived weight gain among LGBTQ+ individuals (*n* = 120) and cisgender and heterosexual-identifying women (*n* = 230), to cisgender and heterosexual-identifying men (*n* = 61). We also explored complementary interview narratives among LGBTQ+ people by employing selective coding strategies.

**Results:**

Study results suggest that LGBTQ+ individuals are likely experiencing uniquely high levels of pandemic-related stress, and secondly, that pandemic-related stress is associated with elevated eating disorder symptoms and higher risk of perceived weight gain. Nearly 1 in 3 participants reported eating disorder symptoms of potentially clinical significance. Social support, but not resilient coping, was found to be protective against increased eating disorder symptoms. Qualitative analyses revealed that LGBTQ+ individuals situated physical exercise constraints, challenging eating patterns, and weight concerns within their pandemic experiences.

**Conclusions:**

Clinicians of diverse specialties should screen for eating disorder symptoms and actively engage patients in conversations about their COVID-19-related weight gain and eating behaviors, particularly with LGBTQ+ -identifying adults.

## Plain English summary

The COVID-19 pandemic has uniquely shaped the mental health of individuals globally. Adults, particularly those identifying as lesbian, gay, bisexual, transgender, and/or queer (LGBTQ+), may be at risk of engaging in disordered eating behaviors (eating disorder symptoms) due to pandemic-related stress. This study explores correlates of eating disorder symptoms and perceived weight gain among adults during the COVID-19 pandemic. We surveyed 411 US-residents, and engaged in follow-up semi-structured interviews with 43 LGBTQ+ -identifying survey respondents to better establish the unique pandemic-related experiences and mental health challenges of LGBTQ+ adults. We quantitatively found that LGBTQ+ individuals experienced uniquely high levels of pandemic-related stress, and secondly, that pandemic-related stress was associated with increased eating disorder symptoms and perceived weight gain. Qualitative narratives complemented quantative findings and suggested that eating concerns, physical exercise constraints, and weight concerns were important to LGBTQ+ people’s perceived health challenges during the pandemic. These findings highlight the need to screen for any persistent eating disorder symptoms or weight concerns, particularly among LGBTQ+ adults.

## Background

The coronavirus disease (COVID-19) pandemic has shaped the mental health of many U.S. residents [1, 2]. Pandemic-related anxiety and stress is likely tied to fear of infection [3] and disruption of routine [4]. Individuals with pre-existing mental health challenges, such as eating disorders (ED), may experience elevated COVID-19 pandemic stress and aggravation of their mental health symptoms [5]. While there is emerging research on ED and weight change during the COVID-19 pandemic, a paucity of research explores the experiences of lesbian, gay, bisexual, transgender, or queer (LGBTQ+) individuals. This study expounds upon correlates of ED symptoms and perceived weight change at months 8–10 of the COVID-19 pandemic among U.S.-residing individuals. In particular, this study employs a convergent mixed-method design to evaluate the influence of pandemic-related stress on perceived weight change and eating disorder symptoms among a community sample of LGBTQ+ -identifying adults.

While several studies suggest differential outcomes based on ED type (e.g., anorexia nervosa vs binge eating) [6], persons with histories of ED may be particularly affected by pandemic-related stressors, exacerbating risk for recurrence or elevated severity of their ED and worsening symptoms [6–12]. Preliminary studies examining disordered eating behaviors (DEB) (e.g., the behaviors often symptomatic of eating disorders, such as fasting or skipping meals) during the COVID-19 pandemic cautioned how lockdown may exacerbate problematic eating behaviors [13–15], and that ED may shape COVID-19 risk [16]. In an Italian community sample collected at two different phases of Italy’s pandemic lockdown, Cecchetto et al. [17] found that depression and anxiety were associated with emotional eating, and that stress was associated with binge eating. Similarly, in a French college sample, Flaudias et al. [13] found that lockdown-related stress and elevated exposure to COVID-19-related media was predictive of dietary restrictions and/or binge eating. For many U.S.-residents, lockdown was not mandated, but encouraged, and it is likely that pandemic-related stress shaped dietary behaviors and DEB in community populations.

Coinciding with reliance on online technologies during the pandemic is the potential risk for elevated exposure to weight-stigmatizing social media messaging [18]. Social media messaging on COVID-19 weight gain (often called the “COVID-15”) ranges from self-acceptance of potential weight gain to imagery that mocks obesity and is overtly weight-stigmatizing [18]. While there is extensive research highlighting the potential risks for weight gain [19, 20], emerging studies suggest that these risks are being realized, with some populations enduring self-reported weight gain during the pandemic [21, 22].

COVID-19 stressors likely intersect with exposure to weight stigma, triggering elevated perceptions of weight gain, and exacerbating DEB [18, 23]. However, individual-level resilience and social support likely offset the potential adverse effects of pandemic stress on unhealthy eating behaviors and risk of negative body image/perceived weight gain during the pandemic [23, 24]. While often examined separately, it is important to consider the influence of pandemic stress on ED symptoms and body weight perceptions simultaneously, as they are likely interrelated and/or mutually reinforcing. It is also important to establish whether individual resilience and/or social support may be protective against DEB or perceived weight gain.

Previous studies have shown that LGBTQ+ identifying young people may experience elevated risk of ED or DEB [25–29], and that disparities in engagement in DEB may be increasing for sexual minority youth [29]. Transgender young people also have been found to experience high prevalence of DEB, tied to experiences of stigma and discrimination [30, 31]. While the literature is mixed regarding the heterogeneity across LGBTQ+ subgroups [25, 26]. LGBTQ+ individuals may be more likely to engage in DEB as a coping mechanism for chronic minority stress, or perceived lack of control in their daily lives, relative to cisgender and heterosexual-identifying peers. Minority stress occurs in response to anti-LGBTQ+ sentiment in the forms of prejudice, discrimination, and harassment [32], and shapes both physical and mental health [33].

Emerging research has documented LGBTQ+ people’s increased vulnerability to severe COVID-related outcomes based on their higher prevalence of comorbidities [34]. Elevated levels of existing mental health challenges among LGBTQ+ individuals likely increases their vulnerability to pandemic-stress [35–37]. Preliminary studies suggest LGBTQ+ people’s health has been uniquely impacted by pandemic mitigation efforts, such that social distancing simultaneously shaped increases in alcohol use and reductions in LGBTQ+ community connections and minority stress [38]. Based on reports of elevated pandemic-related distress among LGBTQ+ people already documented [39], it is imperative to further examine whether pandemic stress is uniquely shaping LGBTQ+ people’s risk for ED and perceptions of weight gain during a pandemic to better inform interventions.

Given prior research suggesting that pandemic-related stress is heightened for LGBTQ+ individuals who likely are already navigating complex minority stressors, we hypothesize that LGBTQ+ adults will report elevated pandemic-related stress when compared with cisgender and heterosexual (cishet) peers (Hypothesis 1). In addition, we hypothesize that an increase in pandemic stress will be (quantitatively) associated with increase in ED symptoms and elevated likelihood of perceived weight gain (Hypothesis 2) in the overall sample. We also anticipate that LGBTQ+ participants will (qualitatively) highlight potential or perceived weight gain and food concerns in their interview narratives detailing the impact of COVID-19 pandemic on their physical health (Hypothesis 3), given prior research suggesting a media and cultural focus on pandemic weight gain.

## Methods

### Procedure

**Fig. 1:**
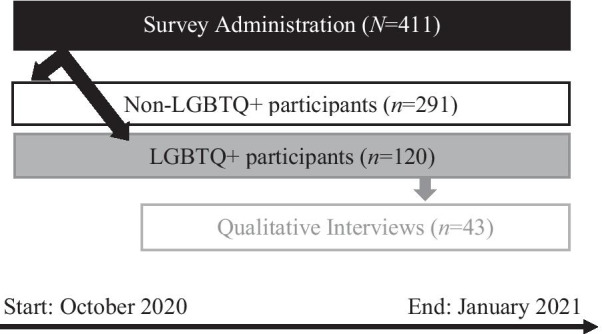
Convergent mixed method design

This study employed a convergent mixed-method design, collecting quantitative survey and qualitative interview data simultaneously (see Fig. 1). Quantitative and qualitative data collection ran between October 2020 and January 2021. We employed a blend of purposive and convenience sampling strategies, targeting university employee and student listservs in three states (Oklahoma, Wyoming, Texas) and local LGBTQ+ social organizations’ social media pages to oversample for LGBTQ+ respondents. Potential participants were invited to participate in a self-administered online survey via secure survey link (Qualtrics™). Survey responses were anonymous, and participants were presented with an electronic consent cover-letter. Participants were informed that on completion of the survey, they would be redirected to submit an email entry to the study’s $25 gift card raffle (four winners were selected). Approximately 490 respondents started the survey, but only 411 were fully completed.

We also simultaneously invited respondents who self-identify as LGBTQ+ to participate in a qualitative interview. Response rate for interviews was 65%, and we interviewed 43 LGBTQ+ identifying participants using a semi-structured interview guide and conducted virtually via Zoom™. Example questions include, “How has the pandemic impacted your physical health?” Interview participants were compensated with a $20 Amazon gift card, and the audio-recorded interviews lasted on average one hour. Participants selected their own pseudonyms to ensure confidentiality of responses. Study procedures were reviewed and approved by University Institutional Review Board.

### Measures

#### Dependent variables

This study uses the Eating Disorder Examination—Questionnaire Short (EDE-QS) [40, 41], which includes 12 items with a response scale ranging from 0 to 3, that were then summed to create an *ED symptoms score* (range = 0–36) (*α* = 0.91). While multivariate analyses treat the score as continuous, we also apply suggested EDE-QS cut-off of 15 points to establish proportion of sample experiencing ED symptoms that are likely clinically significant [41].

We also examine *self-reported weight change*, comparing those who reported no weight change (Reference), to those who report either having lost weight, or having gained weight.

#### Primary independent variable

*Pandemic stress scale* [42] is a composite score based on 5 items (range = 5–25) that asks respondents to consider how worried they have been about the COVID-19 pandemic over the past 14 days. Example items include, “I am worried that I can’t keep my family safe from the virus,” with response options ranging from 1 “not at all” to 5 “extremely” bothered (for full scale, please see the Appendix). This scale was found to have high internal consistency (*α* = 0.91).^1^

#### Key covariates

*LGBTQ*+ *identity* combines information from self-reported sex assigned at birth, gender identity, and sexual identity, to create a 3-category measure comparing cisgender and heterosexual (cishet) women, and LGBTQ+ identifying participants, to cishet men (reference). *Body Mass Index (BMI)* was calculated using self-reported height and weight information (kg/m^2^), excluding individuals with biologically improbable values (BIVs) [43]. *Resilience* is a composite score based on four-items (range = 4–20) (*α* = 0.75) from the Brief Resilient Coping Scale [44]. *Social support* is a composite score measured using the eight-item, modified Medical Outcomes Study Social Support Survey (mMOS-SS) [45] (range = 8–40) (*α* = 0.93).

#### Additional covariates

*Age* is measured in age in years. *Income* is measured using ordinal income brackets ranging from “less than 10,000” (1) to “more than 90,000” (10). *Employment status* compares those who are employed full-time (reference) to those who work part-time, are a student, or currently not working. *Marital status* compares those who are currently married to those who are not married (reference), *Latinx ethnicity* compares those who identify as being Hispanic/Latino/a/x or of Spanish origin to those who identify as non-Hispanic (reference). *Race* compares those who identify as white (reference) to those identifying as non-white. Self-reported *Zip code* is also considered.

### Analytic approach

#### Quantitative analyses

Using Stata 15.1, we first present descriptive statistics, followed by ordinary least squares (OLS) regression results modeling predictors of ED symptoms, and multinomial logistic regression models comparing relative risk of perceived weight gain or weight loss (reference is no weight change) as a function of our key independent variables (LGBTQ+ identity, pandemic stress, and resilience). Baseline models include LGBTQ+ identity only. Model 1 includes LGBTQ+ identity, social support, BMI and resilience in addition to controls (age, income, ethnicity, race, marital status, and employment status). Model 2 adds pandemic stress to model 1 to better assess how pandemic stress may attenuate the relationship between key independent variables and outcomes. All multivariate analyses are clustered by zip code (*n* = 146) to adjust for place-based differences. Significance is based on 95% confidence.

#### Qualitative analysis

All interview audio recordings were transcribed and uploaded into MAXQDA for analysis. The second and third authors conducted three rounds of coding following grounded theory tenets [46], beginning with open coding to identify general patterns of LGBTQ+ people’s stress responses to the pandemic and coping strategies. Next, the coders deployed axial coding to establish connections between and among the codes. Finally, the coders engaged in selective coding to delineate the overarching categories, or domains, which ultimately comprised our final qualitative thematic findings. The primary coders widely agreed on coding decisions, resulting in a 95% intercoder agreement.

## Results

### Quantitative sample

After listwise deletion of cases with missing responses (*n* = 89), the analytic sample includes *N* = 411 U.S. participants predominantly from the Mountain West/Midwest US region, including Oklahoma, Wyoming, and Texas, followed by South Dakota, Utah, Arkansas, and Arizona. Nearly 21% of the sample identified as a man, 74% identified as a woman, and 5% identified as transgender, gender queer, or non-binary. In addition, 71% of the sample identified as exclusively heterosexual, and 29% identified their sexuality as LGBQ+. This resulted in a subsample of cisgender/heterosexual-identifying (cishet) men (*n* = 61, 15%), cishet women (*n* = 230, 56%), and LGBTQ+ -identifying participants (*n* = 120, 29%) (Table 1). The average age of respondents was 28.5 years (*sd* = 11.4, range = 18–86), and only 8% of the sample is over age 50. In addition, 87% identified racially as white, 14% identified ethnically as Latinx, and on average, respondents reported total household incomes in the $50,000 to $59,999 per year income bracket.

**Table 1:** Descriptive statistics of outcomes and key covariates stratified by LGBTQ+ identity

	Everyone	Cishet men	Cishet women	LGBTQ+	*P* value^a^
	*N* = 411	*n* = 61	*n* = 230	*n* = 120	
*Outcomes*					
ED symptoms (EDE-QS) (r = 0–36)	10.47 (8.54)	7.61 (7.50)	10.67 (8.67)	11.53 (8.54)	0.003
Score of 15 or higher^b^	30%	18%	29.6%	38%	< 0.001
Perceived weight change					
Gained	45%	28%	43%	57%	< 0.001
Lost	20%	21%	20%	20%	< 0.001
Stayed the same	35%	51%	37%	23%	< 0.001
*Primary independent variable*					
Pandemic stress (r = 5–25)	14.9 (5.9)	11.6 (5.5)	14.2 (11.0)	17.9 (5.4)	< 0.001
*Key covariates*					
BMI (r = 15.7–62.0)	26.7 (6.7)	25.9 (5.0)	26.2 (6.4)	28.1 (7.9)	0.052
Social support (r = 8–40)	30.1 (8.4)	27.3 (9.8)	31.1 (8.4)	29.7 (7.2)	0.064
Resilience (r = 4–20)	12.6 (3.4)	13.8 (3.0)	12.5 (3.4)	12.3 (3.4)	0.004

Data are from primary data related to COVID-19 pandemic experiences collected between October 2020 and January 2021

Cishet, cisgender and heterosexual identifying; r, range; EDE-QS, Eating Disorder Examination—Questionnaire Short; BMI, Body Mass Index

^a^*p *values reflect results from unadjusted comparisons between LGBTQ+ identifying participants and cisgender/heterosexual-identifying men using independent two-tailed t-tests for means and chi-squared for frequencies

^b^Suggested screening cut-off proposed by Prnjak et al. [41] that may suggest clinically significant impairment associated with ED symptoms

### Quantitative results

Descriptive statistics suggest that cishet women (mean = 10.7, *sd* = 8.7) and LGBTQ+ identifying respondents (mean = 11.5, *sd* = 8.5) reported more ED symptoms than cishet men (mean = 7.61, *sd* = 7.5) (Table 1). Applying suggested EDE-QS cut-offs for clinical significance [41], approximately 30% of our sample at or above their most sensitive cut-off of 15 points. Among LGBTQ+ participants, 38% scored 15 points or higher on the EDE-QS, suggesting that more than 1 out of 3 LGBTQ+ persons experienced ED symptoms of possibly clinical significance. Furthermore, greater proportions of LGBTQ+ participants (57%) and cishet women (43%) reported that they have gained weight during the pandemic relative to cishet men (28%, *p* < 0.001) (Table 1). LGBTQ+ respondents also scored approximately 3.0 points higher on pandemic stress, and 1.5 points lower on resilient coping, relative to cishet men (*p* < 0.01) (Table 1).

OLS regression results suggest that when excluding pandemic stress, LGBTQ+ and cishet women had elevated ED symptoms relative to cishet men, holding age, Hispanic ethnicity, race, income, marital status, and employment status constant (Table 2, model 1). However, when pandemic stress is added to the model, LGBTQ+ identity becomes non-significant (model 2). Every one unit increase in pandemic stress score was associated with 0.23 increase in ED symptoms score, holding all else constant (OLS coefficient = 0.23[95% Confidence Interval (CI) = 0.11–0.35], *p* < 0.001) (model 2). BMI is also associated with ED symptoms, with a one unit increase in BMI being associated with an 0.35 increase in ED symptoms, holding all else constant (p < 0.001) (model 2). While social support was found to be associated with reduced ED symptoms in model 1, when pandemic stress is considered (model 2), the association is no longer significant. Similarly, resilient coping was not found to be significantly associated with ED symptoms.

**Table 2:** OLS regression results: ED symptoms (EDE-QS)

ED symptoms	Baseline	Model 1	Model 2
Cishet men (reference)			
Cishet women	3.07 [1.44–4.69] (0.82)***	3.05 [1.47–4.62] (0.80)***	2.52 [0.86–4.18] (0.84)**
LGBTQ+ participants	3.93 [1.81–6.04] (1.07)***	2.19 [0.32–4.05] (0.94)*	0.94 [− 1.08–2.95](1.02)
BMI		0.35 [0.26–0.45] (0.05)***	0.33 [0.24–0.42] (0.05)***
Social support		− 0.15 [− 0.23 to − 0.08] (0.05)***	− 0.15 [− 0.23 to − 0.07] (0.04)***
Resilience		− 0.09 [− 0.32 to 0.16] (0.12)	− 0.05 [− 0.30 to 0.20] (0.13)
Pandemic stress			0.23 [0.11–0.35] (0.06)***
N	411	411	411
R-squared	0.02	0.16	0.18
AIC	2925	2883	2875

Data are from primary data related to COVID-19 pandemic experiences collected between October 2020 and January 2021. Coefficients presented, 95% confidence intervals in brackets, robust standard errors in parentheses. Models 1 & 2 also adjusts for age, Hispanic ethnicity, race, income, marital status, and employment status

Cishet, cisgender and heterosexual-identifying; AIC, Akaike information criterion; EDE-QS, Eating Disorder Examination—Questionnaire Short; BMI, Body Mass Index. Results are clustered by US Zip Code (*n* = 146)

+ *p* < 0.01, *p < 0.05, **p < 0.01, ***p < 0.001

Multinomial logistic regression results suggest that the relative risk of reporting having gained weight (relative to reporting no weight change) is elevated at a factor of 3.82 (95% CI = 1.90–7.70) for LGBTQ+ participants relative to cishet men (holding controls constant) (Table 3, model 1a, *p* < 0.001). Specifically, LGBTQ+ respondents have higher relative risk of reporting having gained weight (in comparison to reporting no weight change) relative to cishet men (ceteris paribus). LGBTQ+ participants also had expected elevated relative risk of reporting having lost weight (versus reporting no weight change) relative to cishet men, holding all else constant (Relative Risk Ratio [RRR] = 2.57[95% CI = 1.09–6.09], *p* < 0.05) (model 1b). When pandemic stress is considered, LGBTQ+ respondents' elevated relative risk of reporting gained weight (versus no weight change) relative to cishet men holds (RRR = 2.84[95% CI = 1.33–6.08], *p* < 0.01) (model 2a). Pandemic stress is associated with elevated relative risk of reporting having gained weight (compared to no weight change), where a one unit increase in pandemic stress increases the relative risk of reporting having gained weight versus no weight change by a factor of 1.06 (95% CI = 1.03–1.11), holding all else constant (*p* < 0.001) (model 2a). We failed to detect significant associations between social support and resilient coping with perceived weight change in any model.

**Table 3:** Multinomial logistic regression results: perceived weight change (reference is no weight change)

Perceived weight change	Baseline	Baseline	Model 1a	Model 1b	Model 2a	Model 2b
	Weight-gain	Weight-loss	Weight-gain	Weight-loss	Weight-gain	Weight-loss
Cishet men (reference)						
Cishet women	2.17 [0.96–4.89] (0.90)+	1.31 [0.70–2.44] (0.42)	2.29 [0.93–5.63] (1.05)+	1.76 [0.93–3.35] (0.58)+	2.02 [0.82–5.01] (0.99)	1.64 [0.85–3.15] (0.54)
LGBTQ+	4.43 [2.25–8.70] (1.53)***	2.04 [0.93–4.47] (0.82)+	3.82 [1.90–7.70] (1.37)***	2.57 [1.09–6.09] (1.13)*	2.84 [1.33–6.08] (1.10)**	2.15 [0.92–4.77] (0.90)+
BMI			1.19 [1.13–1.26] (0.03)***	1.12 [1.05–1.19] (0.04)***	1.18 [1.12–1.25] (0.03)***	1.12 [1.05–1.19] (0.04)***
Social support			1.00 [0.97–1.03] (0.01)	0.97 [0.94–1.00] (0.02)+	1.02 [0.99–1.05] (0.02)	0.97 [0.94–1.00] (0.02)
Resilience			0.96 [0.88–1.03] (0.04)	1.00 [0.91–1.11] (0.05)	0.97 [0.90–1.05] (0.04)	1.01 [0.91–1.12] (0.05)
Pandemic stress					1.06 [1.03–1.11] (0.02)**	1.04[0.98–1.11] (0.04)
*N*	411		411		411	
Pseudo R-squared	0.02		0.10		0.11	
AIC	857		829		827	

Data are from primary data related to COVID-19 pandemic experiences collected between October 2020 and January 2021. Relative risk ratios presented, 95% confidence intervals in brackets, robust standard errors in parentheses. Model 1 and 2 also adjusts for age, Hispanic ethnicity, race, income, marital status, and employment status. Results are clustered by US Zip code (*n* = 146)

BMI, Body Mass Index; AIC, Akaike information criterion; Cishet, cisgender and heterosexual identifying

+*p* < 0.01, *p < 0.05, **p < 0.01, ***p < 0.001

### Qualitative subsample

The final analytic qualitative subsample included *n* = 43 LGBTQ+ -identified people aged 18+, with an average age of 28 years old. Table 4 presents detailed sociodemographic features of the qualitative subsample.

**Table 4:** Descriptive statistics of the LGBTQ+ participants from the qualitative subsample

Sociodemographic variables (qualitative subsample *n* = 43)	Sample size/percentage n (%)	Mean/SD
Age (range 19–59)		27.7/9.2
*Sexual identity*		
Lesbian	8 (19)	
Gay	7 (16)	
Bisexual	15 (35)	
Queer	3 (7)	
Pansexual	5 (12)	
Asexual	2 (5)	
Expansive sexuality/unlabeled	3 (7)	
*Gender identity*		
Cisgender women	27 (63)	
Cisgender men	10 (23)	
Nonbinary	3 (7)	
Transgender woman	1 (2)	
Queer	2 (5)	
*Race/ethnicity*		
White	34 (79)	
Bi or multiracial	3 (7)	
Latino/a or Hispanic	5 (12)	
Asian American	1 (2)	
*Regional identification*		
Rural	19 (44)	
Urban	9 (21)	
Suburban	15 (35)	
*Social class status*		
Working class	19 (44)	
Middle class	19 (44)	
Upper middle class	5 (12)	

Data are from primary data related to COVID-19 pandemic experiences collected between October 2020 and January 2021

### Qualitative results

Interviews with LGBTQ+ people revealed that the pandemic is creating complex challenges for them to navigate in terms of their weight and eating behaviors. Participants highlighted how disruptions to routine acts as a barrier to both ongoing and prospective exercise routines (*physical exercise constraints*), and pandemic-related stress and boredom may be shaping unhealthy eating habits (*eating patterns*). LGBTQ+ participants, through detailing their *physical exercise constraints* and unhealthy *eating patterns,* ultimately discussed that they have gained weight throughout the pandemic and conceptualized weight gain as adversely influencing their physical health (*weight concerns*) as well as their broader mental and emotional wellbeing.

#### Physical exercise constraints

As a result of widespread pandemic-related closures, virtual workplace transitions, and social distancing mandates, all participants discussed to some extent how these significant changes to their everyday lives impacted their physical movement routines. From disruptions to exercise regimens to constraints to feeling more shackled to technology, LGBTQ+ people discussed the harmful ways that the pandemic created barriers to physical activity. Logan (White cisgender bisexual man) explicitly attributed his loss of feelings of physical fitness and exercise routine to pandemic-related changes such as the shift from in-person activities to all interactions occurring virtually: “I think my physical health has gotten a little worse because before the pandemic, I was hitting the gym pretty much every day. And when the pandemic hit, I went from going to the gym to not going to the gym and spending all my time on Zoom. And so I have gotten a little out of shape.” Similarly, Layla (White transgender unlabeled woman) expressed physical-health related challenges tied to the use of her legs due to a more sedentary lifestyle within the pandemic and not being able to maintain mobility and fitness: “I haven't been able to go anywhere and exercise, so I'm very much out of shape. That's definitely there.”

#### Eating patterns

In addition to physical exercise constraints, LGBTQ+ participants also highlighted how the pandemic shaped more challenging, stress-related, and unhealthy-categorized eating patterns. In the case of Manuel (Latino cisgender gay man), exercise became a routine addition within the pandemic, but this positive was tempered by his framing of excessive eating as a way to cope with boredom and being at home more often: “I definitely put on quite a bit of weight and not eating exactly the healthiest. I have started exercising. That's kind of a positive, but yeah, mostly just the foods that I'm eating and like the excess of food… I'm eating now because I'm bored. I have nothing else to do where I'm constantly snacking on something just because I’m home.” Further intersecting with the challenges participants highlighted with pandemic-induced constraints to exercise, Allison (White cisgender lesbian woman) conceptualized her changing eating patterns (and self-identified weight gain) as a type of response to not being able to go to the gym anymore and being more homebound: “I was going to the gym fairly regularly and then the pandemic hit and I just canceled my gym membership and I haven't been back since, and I, you know, I eat a lot more ice cream than I should because I'm at home, so definitely I gained weight.”

#### Weight concerns

Often in combination with LGBTQ+ people’s concerns with pandemic-influenced physical exercise constraints and eating patterns framed as unfavorable, participants also expressed heightened concerns with weight fluctuations that they deemed harmful for their physical health. An exemplar of the intersecting challenges of exercise constraints, eating patterns, and weight concerns is Valeria (Latina cisgender pansexual woman), who attributed her physical health struggles to pandemic disruptions like a lack of routine schedule: “I found myself snacking more than usual. Some days I would overeat, other days I would barely eat anything. The major concern has always been my weight. So that's something I'm looking forward to, like getting back on track with the whole, exercising and trying not to snack as much as before.” For some participants, weight concerns translated into potentially more serious health challenges as defined by a health care provider, as in the case of Greg (White cisgender gay man): “We had goals we were trying to meet. And then that fell apart and the gym closed. And I fell off the wagon and wasn't able to do the workout routine that I had started. So I definitely gained weight during the pandemic. And just a couple of weeks ago, I met with my primary care doctor who was like, ‘You need to get back on this routine.’".

## Discussion

This study explores ED symptoms and weight change perceptions at months 6–8 of the COVID-19 pandemic in the U.S., and has three major findings; first, that LGBTQ+ individuals are likely experiencing uniquely high levels of pandemic-related stress compared to cisgender/heterosexual men (hypothesis 1 supported), and secondly, that pandemic-related stress is strongly associated with ED symptoms and perceived weight gain (hypothesis 2 supported). Third, LGBTQ+ people’s interview narratives underscore complex pandemic-related health challenges, ranging from exercise constraints and disrupted eating patterns, to weight fluctuation concerns (hypothesis 3) that converged with the quantitative findings regarding LGBTQ+ people’s elevated ED symptoms and perceived weight gain. While quantitative results focused on the influence of pandemic stress, qualitative narratives added additional understanding of how individuals associate being homebound (or spending a greater proportion of time at home) with disruptions to eating behavior and perceived weight gain.

Many U.S. residents, similar to peers globally, have endured unique pandemic-related stress and constraints, and our study supports prior research suggesting pandemic-specific stress and disruption to routine is likely to exacerbate pre-existing ED symptoms and/or result in engagement in new DEBs. We found that nearly 30% of our sample may be experiencing ED symptoms of clinical significance, with 38% of LGBTQ+ individuals potentially experiencing clinically significant impairment associated with ED symptoms. While purposive sampling of LGBTQ+ community members may be skewing results toward higher ED symptoms in this sample, approximately 18% of cisgender and heterosexual identifying men also scored 15 or higher.

While it is possible that, given the short time frame of the EDE-QS, that ED symptoms and weight-related concerns will subside post-pandemic, our study confirms that LGBTQ+ individuals are experiencing elevated pandemic-related stress that may be detrimental to their physical and mental wellbeing. LGBTQ+ individuals are likely navigating identity-related minority stressors that are exacerbated or interlocking with pandemic-specific stressors that lead to greater pandemic related stress levels [35]. For example, LGBTQ+ young people are at risk of experiencing elevated interpersonal trauma in their home environments, as many LGBTQ+ individuals at risk of exposure to identity-related stigma and discrimination, including domestic violence, from family members [37]. These LGBTQ+ identity related *minority stressors* can have a detrimental effect on behavioral and mental health outcomes [47], over and above effect of universal stressors (e.g., the COVID-19 pandemic) [48].

Our findings suggest a strong association between pandemic stress and ED symptoms. While it is likely that the association is bidirectional or mutually reinforcing (e.g., individuals with ED symptoms may be more likely to report pandemic-related stress), interview narratives suggest that LGBTQ+ individuals perceive the pandemic to be adversely impacting their eating and exercise routines, leading to potential or perceived weight gain. While respondents may not have experienced actual weight gain, over-evaluation of body weight and weight concerns are known risk factors for ED [49]. Higher BMI was found to be predictive of elevated ED symptoms and perceived weight gain, suggesting that body weight is also an important contribution to people’s pandemic experiences. Many interview participants suggested that they were eating more frequently, and poorly, due to a combination of perceived stress and boredom due to being homebound. Prior studies suggest that cognitive factors such as boredom and rumination are associated with mental health challenges such as symptoms of anxiety and depression [50]. Future studies should examine the role of these cognitive factors when investigating the long-term effects of the pandemic.

LGBTQ+ interview narratives convergent with the quantitative findings and with respondents perceiving their weight fluctuations to have real adverse effects on their physical health. Notably, social support was associated with alleviating symptoms of ED, suggesting that perceived social support may be a protective factor against ED resulting from pandemic-related stress. However, resilience did not emerge as a significant protective factor; resilience is a dynamic process wherein one bounces back with positive adaptations after facing an adverse event(s) [51]. It may be that on-going COVID-19 related stressors continue to influence the dynamic process of resilience building, with social support playing an influential role in protecting against pandemic stress [52].

However, a major limitation of this study is that data are cross-sectional and cannot speak to processes over time, such as actual weight gain or change in ED symptoms across the pandemic. In addition, cisgender women and LGBTQ+ identifying persons were more likely to complete the survey, and therefore results under consider the experiences of cisgender men. Furthermore, we did not have large enough subsamples of LGBTQ+ respondents to engage in more detailed examination of LGBTQ+ heterogeneity. In addition, there is likely racial and ethnic disparities in pandemic-related stress, and its association with ED symptoms and perceptions of weight gain, which we were unable to fully assess due to sample size constraints.

## Conclusions

Our study highlights that pandemic-related stress is likely elevating community members’ ED symptoms, and contributing to perception and concerns around perceived weight gain. While many respondents were hopeful that they would be able to “get back to'' healthy eating and exercise routines at some point in the future (e.g., when pandemic restrictions and social isolation ends), future studies should consider the potentially long-term effects of the pandemic on individuals’ perceived or actual weight gain, and ED symptoms. To ensure proper monitoring, health care practitioners should widely screen for ED symptoms among people of all genders and sexualities, and actively engage in conversations with their patients about their perceived weight gain and eating behaviors.

## Data Availability

The data that support the findings of this study are available on request from the corresponding author.

## Abbreviations

COVID-19: Coronavirus disease
ED: Eating disorder
DEB: Disordered eating behaviour
LGBTQ+: Lesbian, gay, bisexual, transgender, and/or queer
cishet: Cisgender and heterosexual
BMI: Body mass index
OLS: Ordinary least squares
sd: Standard deviation
95%-CI: 95% Confidence interval
U.S.: United States

## Footnotes

1 In sensitivity analyses (not shown) testing other measures of pandemic-related stress that consider additional conceptualizations of pandemic-related stress, such as traumatic stress, and obsessive COVID-19 checking behaviors, results did not change substantively.
